# Long non-coding RNA CASC2 improved acute lung injury by regulating miR-144-3p/AQP1 axis to reduce lung epithelial cell apoptosis

**DOI:** 10.1186/s13578-018-0205-7

**Published:** 2018-02-26

**Authors:** Hongbin Li, Huijuan Shi, Min Gao, Ning Ma, Rongqing Sun

**Affiliations:** Department of Critical Care Medicine, The First Affiliated Hospital of Zhengzhou University, No.1 Jianshe East Road, Zhengzhou, 450052 Henan China

**Keywords:** lncRNA CASC2, Acute lung injury, Lung epithelial cells apoptosis, miR-144-3p, AQP1

## Abstract

**Background and objective:**

Apoptosis of lung epithelial cell is implicated in the pathogenesis of acute lung injury (ALI). To study the protective effect and mechanism of cancer susceptibility candidate 2 (CASC2) on reducing lung epithelial cell apoptosis after LPS inducing acute lung injury in mice.

**Methods and results:**

The ALI mice model was performed by intratracheally instilling with lipopolysaccharide (LPS). The CASC2 expression detected by quantitative real-time polymerase chain reaction was significantly decreased in LPS-induced A549 cell and ALI mice model. LPS induced A549 cell apoptosis, while transfection with pcDNA-CASC2 reversed the increased cell apoptosis, suggesting overexpression of CASC2 inhibited LPS-induced A549 cell apoptosis. In addition, we found that miR-144-3p expression were opposite to CASC2, while Aquaporin-1 (AQP1) expression was opposite to miR-144-3p in LPS-induced A549 cell and ALI mice model. The RNA immunoprecipitation and RNA pull-down assay demonstrated that CASC2 could function as a miR-144-3p decoy. The luciferase reporter assay revealed that AQP1 was a target of miR-144-3p in A549 cell. And then, further in vitro studied showed that CASC2 controlled AQP1 expression by regulating miR-144-3p, and LPS induced A549 cell apoptosis by regulating CASC2/miR-144-3p/AQP1 axis. At last, after injection with lentivirus-expressing CASC2 or control lentivirus, the mice were intratracheally instilled with LPS. Comparing to the mice injected with pcDNA, the mice injected with pcDNA-CASC2 had a significantly reduced lung wet–dry weight ratio.

**Conclusions:**

Long non-coding RNA CASC2 improved acute lung injury by regulating miR-144-3p/AQP1 axis to reduce lung epithelial cell apoptosis.

## Instruction

Acute lung injury (ALI) is a life-threatening syndrome that can cause acute hypoxemic respiratory failure, which is characterized by diffuse alveolar damage, increased alveolar capillary membrane permeability, edema, excessive pulmonary inflammation and apoptosis of alveolar epithelial cells [1]. Studies have shown that excessive apoptosis of type II alveolar epithelial cells can injury the epithelial barrier thus lead to ALI [2, 3]. Lipopolysaccharide (LPS) is the component of the cell wall of Gram-negative bacteria, which is closely related to alveolar epithelial cell apoptosis [4–6]. Thus, we used LPS to induce apoptosis of alveolar epithelial cells.

Aquaporin-1 (AQP1) is protein that functioned as water transport across cell membranes that mainly expressed in alveolus capillary endothelial cells. A number of studies have shown that AQP1 might play an important role in the pathogenesis of ALI, and the decreased lung AQP1 expression was closely related to the development of ALI, while the upregulated the expression of AQP1 could ameliorate ALI [7, 8].

Recent evidence showed that microRNAs (miRNAs) played an important regulatory role in the development of ALI [9, 10]. For example, Tao [11] and Tang [12] reported that overexpression of miR-454 and miR-126-5p may alleviate ALI. Li et al. [5] found that miR-181a inhibition can significantly protected mice from LPS-induced ALI. These studies indicated that miRNAs might be a potential candidate for the therapy against ALI. MiR-144-3p was a tumor suppressive miRNA in many cancer cells [13, 14], while the expression and the role of miR-144-3p in LPS-induced ALI and lung epithelial cells has not been reported. In addition, bioinformatics software predicted AQP1 was one of the putative target genes of miR-144-3p. Thus, this study raised a question regarding whether miR-144-3p participated in LPS-induced ALI and lung epithelial cells apoptosis by regulating AQP1.

Long noncoding RNA cancer susceptibility candidate 2 (CASC2) has been demonstrated as playing crucial regulatory role in many cancers, such as lung adenocarcinoma, thyroid carcinoma, and hepatocellular carcinoma, etc. However, the expression of CASC2 in ALI and molecular mechanisms underlying CASC2-mediated ALI remain unknown. Bioinformatics software predicted that there were binding sites between CASC2 and miR-144-3p. Thus, we speculated that CASC2 might be involved in ALI by regulating miR-144-3p/AQP1 axis to affect lung epithelial cell apoptosis.

In this study, we first assessed the CASC2 expression in LPS-induced ALI mice and A549 cell. Further experiments were conducted to investigate the biological regulation function of CASC2 with respect to the miR-144-3p and AQP1 expression and cell apoptosis. Additionally, mechanism analysis revealed that CASC2 might function as a ceRNA to regulate AQP1 expression by sponging miR-144-3p, thus affecting LPS-induced lung epithelial cell apoptosis and playing a critical role in the pathobiology of ALI.

## Methods

### Acute lung injury mice model

The BALB/c mice were housed in a room maintained at 25 °C with a light/dark cycle of 12 h/12 h, and then they were randomly divided into two groups (n = 10/group): The control group and LPS group. The ALI mice model was performed by intratracheally instilling with 10 μg LPS in 50 μL of PBS, and the control mice were given an equal volume of PBS. Six hours after the infusion of LPS or PBS, the mice were sacrificed, and lung tissues were harvested for RT-qPCR and western blot. All experimental procedures were approved by the National Institutes of Health Guidelines for the Care and Use of Laboratory Animals.

### Measurement of wet-to-dry ratio of the lungs

Thirty minutes before LPS treatment, the BALB/c mice were injected with 100 μL lentivirus-expressing CASC2 or the control lentivirus (MOI = 5 * 10^7^ TU/mL, GeneChem, Shanghai, China) by tail vein, and then treated with LPS as described in ALI mice model. At 6 h after treatment with LPS, mice were euthanized, the right lung was then removed for detection of mRNA and protein, and determination of wet weight. Subsequently, the lungs were incubated at 60 °C for 3–4 days to remove all moisture, then the dry weight was measured and the ratio of wet-to-dry weight calculated.

### Cell culture and transfection

The human lung adenocarcinoma A549 cell line was purchased from Cell Bank of Type Culture Collection of Chinese Academy of Sciences (Shanghai, China). Cells were grown in DMEM (Sigma-Aldrich) supplemented with 5% FBS (HyClone, Logan, USA) and antibiotics (penicillin, 100 U/mL; streptomycin 100 μg/mL) in a 5% CO_2_ atmosphere at 37 °C. MiR-144-3p inhibitor/mimic, pcDNA-CASC2 and their respective negative control/vector was transfected or co-transfected into A549 cells by Lipofectamine 2000 according to the instructions. After transfection for 48 h, the cell was collected and used to detect mRNA and protein expression and cell apoptosis.

### Quantitative real-time polymerase chain reaction (RT-qPCR)

Total RNA samples were extracted from lung tissue or A549 cells using Trizol (Invitrogen) according to the manufacturer’s instructions. The Taqman microRNA Reverse Transcription Kit and Taqman Universal Master Mix II with the TaqMan MicroRNA Assay of miRNAs (Applied Biosystems, Foster City, USA) were used for testing the miR-144-3P expression level. The level of AQP1 and CASC2 was calculated relative to internal control using the 2^−ΔΔCt^ method using real-time PCR system according to manufacturer’s instructions in SYBR green master mix (Applied Biosystems).

### RNA immunoprecipitation (RIP)

RNA immunoprecipitation assay was performed by the Magna RIP RNA-Binding Protein Immunoprecipitation Kit (Millipore, Billerica, MA, USA) and the AGO2 antibody according to the manufacturer’s protocol. The AGO2 was detected by immunoprecipitation-western, and RT-qPCR detected CASC2 and miR-144-3p in the precipitates. The IgG antibody group as control.

### Western blot analysis

Tissue and cells were collected and lysed in protein lysis buffer. Proteins samples (30 μg) were separated on SDS-12% PAGE and then PAGE transfer onto PVDF membranes (Thermo, USA). Primary mouse monoclonal antibodies against AQP1 and β-actin (Abcam, UK), and secondary antibody peroxidase-conjugated rabbit anti-IgG (Sigma) were used in western blot analysis.

### Apoptosis by flow cytometry assay

Annexin V/PI staining was performed according to previous procedures [15]. Cells from different groups were collected, centrifuged at 1500×*g* for 5 min, and washed with PBS for three times. 1× Annexin V binding buffer was added to make a final concentration of 2 × 10^5^/mL. Annexin-V and PI (propidium iodide) solution (100 μg, 1 μg/ml) were added for staining at room temperature for 15 min, then flow cytometry was used to evaluate cell apoptosis. The apoptotic cells were detected by flow cytometry (FACS 420, BD Biosciences, USA). Percentage of apoptosis rate (%) = (number of apoptotic cells/number of all cells) × 100%.

### Luciferase reporter assay

The 3′UTR of AQP1 including conserved binding sites for miR-144-3p was amplified from human cDNA by PCR. A mutant 3′UTR fragment of AQP1 which the mutations was in conserved binding sites for miR-144-3p, was also generated. The fragments including the 3′UTR regions (3′UTR-WT) or mutant 3′UTR regions (3′UTR-Mut) of AQP1 were inserted into vector p-Luc-UTR. Then the miR-144-3p mimic/inhibitor and their respective control were transfected into AQP1-overexpressing A549 cells, respectively. After 24 h, cells were collected, and the firefly luciferase activities were determined using a luciferase reporter assay system (Promega, WI) according to the manufacturer’s instructions.

### RNA pull down

The RNA precipitation assay was performed to determine whether CASC2 is coupled with the RISC complex, using synthesized CASC2 as a probe and then detected AGO2 from the precipitation complex using western blot and miR-144-3p using qRT-PCR. The biotin-labeled lncRNA-CASC2 RNA was transcribed in vitro with the Biotin RNA Labeling Mix (Roche) and T7 RNA polymerase (Roche), treated with RNase-free DNase I (Roche), recycled with QIA quick Nucleotide Removal Kit (Qiagen) and purified with the RNeasy Mini Kit (Qiagen). Loc285194 was also cloned as a negative control and used in precipitation experiments for comparison. A549 cell proteins were mixed with biotin-labeled lncRNA-CASC2 RNAs incubated at 4 °C for 1 h. The streptavidin agarose beads (Invitrogen) were added to each binding reaction and incubated at room temperature for 1 h. Western blot was performed to detect AGO2, and the three group of precipitates were used for detecting miR-144-3p expression by RT-PCR according to the standard procedures.

### Statistical analysis

The SPSS 17.0 software (SPSS Inc., USA) was applied for statistical analyses. All experiments were repeated three times, and all data were presented as mean ± standard deviation. The differences between groups were assessed by Student’s t test with a significance level of P < 0.05.

## Results

### Decreased expression of CASC2 in ALI mice

**Fig. 1:**
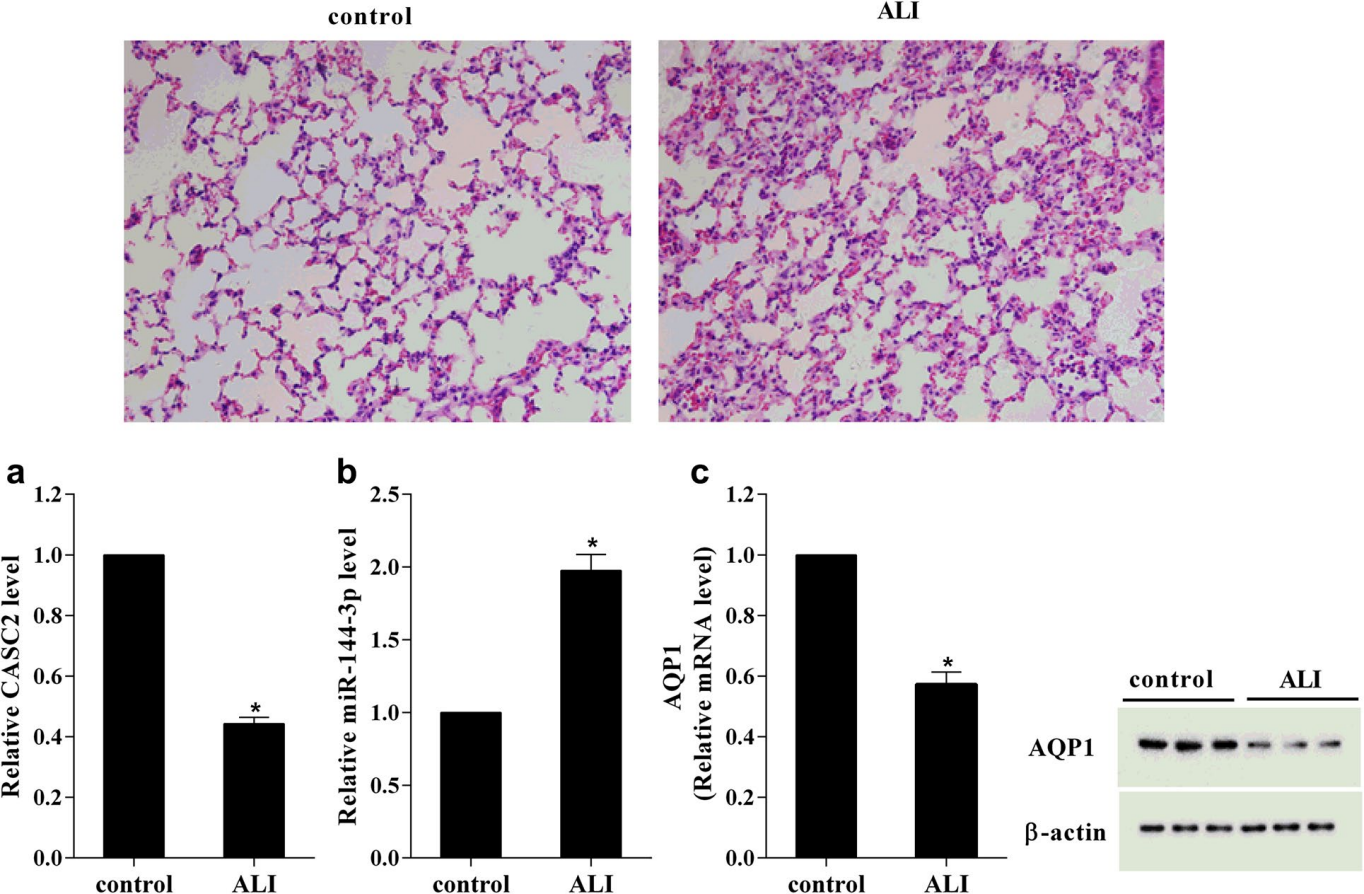
The expression level of CASC2 was decreased in ALI mice. The right lung tissues were respectively isolated from control mice and ALI mice. **a** The CASC2 expression was significantly decreased in ALI mice. **b** The miR-144-3p expression was significantly increased in ALI mice. **c** The AQP1 mRNA and protein expressions were both significantly decreased in ALI mice. *P < 0.05 vs control

To investigate the expression of CASC2, miR-144-3p and AQP1 in ALI, the ALI mice model was built by intratracheally instilling with LPS. And then RT-qPCR and western blot were used to detect the mRNA and protein expression, respectively. H&E staining of the lung tissue of control mice and ALI mice model was shown in Fig. 1. As shown in Fig. 1a, comparing to the control mice, the CASC2 expression in lung tissue of ALI mice was significantly decreased. While the miR-144-3p expression was obviously increased in lung tissue of ALI mice, which was opposite to CASC2 (Fig. 1b). In addition, the AQP1 mRNA and protein were both reduced in lung tissue of ALI mice, which was opposite to miR-144-3p (Fig. 1c). These data suggested that, ALI mice had an increased CASC2 and AQP1 expression, and a decreased miR-144-3p expression.

### Decreased expression of CASC2 in LPS-induced A549 cell

**Fig. 2:**
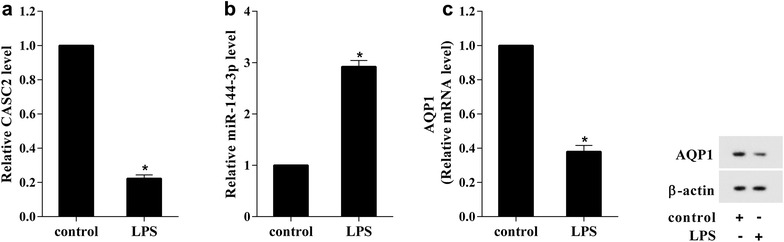
The expression level of CASC2 was decreased in LPS-induced A549 cell. LPS-induced the Human Type II Alveolar Epithelial Cell Line (A549) be as the experimental model in vitro. **a** The CASC2 expression was significantly decreased in LPS-induced A549. **b** The miR-144-3p expression was significantly increased in LPS-induced A549. **c** The AQP1 mRNA and protein expressions were both significantly decreased in LPS-induced A549. *P < 0.05 vs control

As we have known the expression of CASC2, miR-144-3p and AQP1 in ALI, their expression in vitro model of ALI were also detected by RT-qPCR and western blot. The A549 cell induced by LPS as in vitro model of ALI, as Fig. 2a showed that CASC2 expression was obviously decreased in LPS-induced A549 cell. The miR-144-3p expression was significantly increased in LPS-induced A549 cell, which was opposite to CASC2 (Fig. 2b). The AQP1 mRNA and protein expression were both decreased which was opposite to miR-144-3p in LPS-induced A549 cell (Fig. 2c). These data suggested that, LPS could inhibit CASC2 and AQP1 expression, and upregulated miR-144-3p expression in A549 cell.

### Overexpression of CASC2 inhibited LPS-induced A549 cell apoptosis

**Fig. 3:**
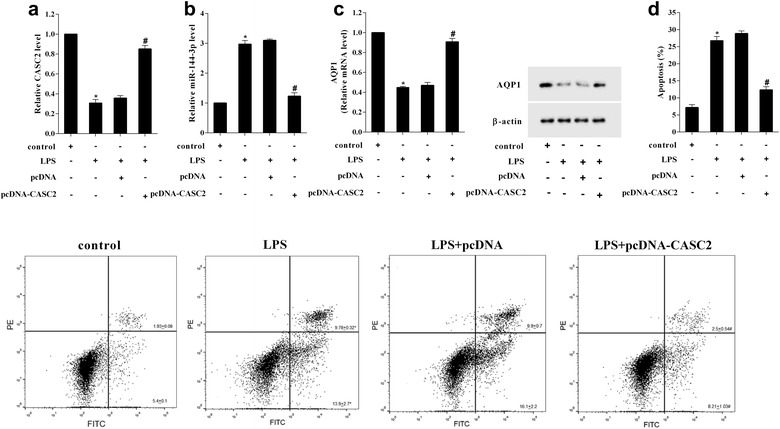
Overexpression of CASC2 inhibited LPS-induced A549 cell apoptosis. To investigate the effect of CASC2 on LPS-induced A549 cell apoptosis, the cell transfected with pcDNA-CASC2 and its control vector, respectively, and then induced by LPS. **a** The pcDNA-CASC2 transfection reversed the LPS-reduced CASC2 expression in A549 cell. **b** The pcDNA-CASC2 transfection reversed the LPS-increased miR-144-3p expression in A549 cell. **c** The pcDNA-CASC2 transfection reversed the LPS-reduced AQP1 expression in A549 cell. **d** The pcDNA-CASC2 transfection reversed the LPS-induced cell apoptosis. *P < 0.05 vs control, ^#^P < 0.05 vs pcDNA

To investigate the effect of CASC2 on LPS-induced A549 cell apoptosis, the A549 cell was transfected with pcDNA-CASC2 and its control vector (pcDNA), and then induced by LPS. The CASC2 expression was inhibited by LPS, but transfection of pcDNA-CASC2 reversed its expression in A549 cell (Fig. 3a). Meanwhile, the miR-144-3p expression was upregulated by LPS, but transfection of pcDNA-CASC2 inhibited its expression in A549 cell (Fig. 3b). The AQP1 mRNA and protein expression were also inhibited by LPS, but transfection of pcDNA-CASC2 reversed its expressions in A549 cell (Fig. 3c). And then, the flow cytometry results showed that LPS induced A549 cell apoptosis, but transfection of pcDNA-CASC2 reduced the apoptosis, which suggested that overexpression of CASC2 inhibited LPS-induced A549 cell apoptosis (Fig. 3d).

### CASC2 functioned as a decoy for miR-144-3p in A549 cell

**Fig. 4:**
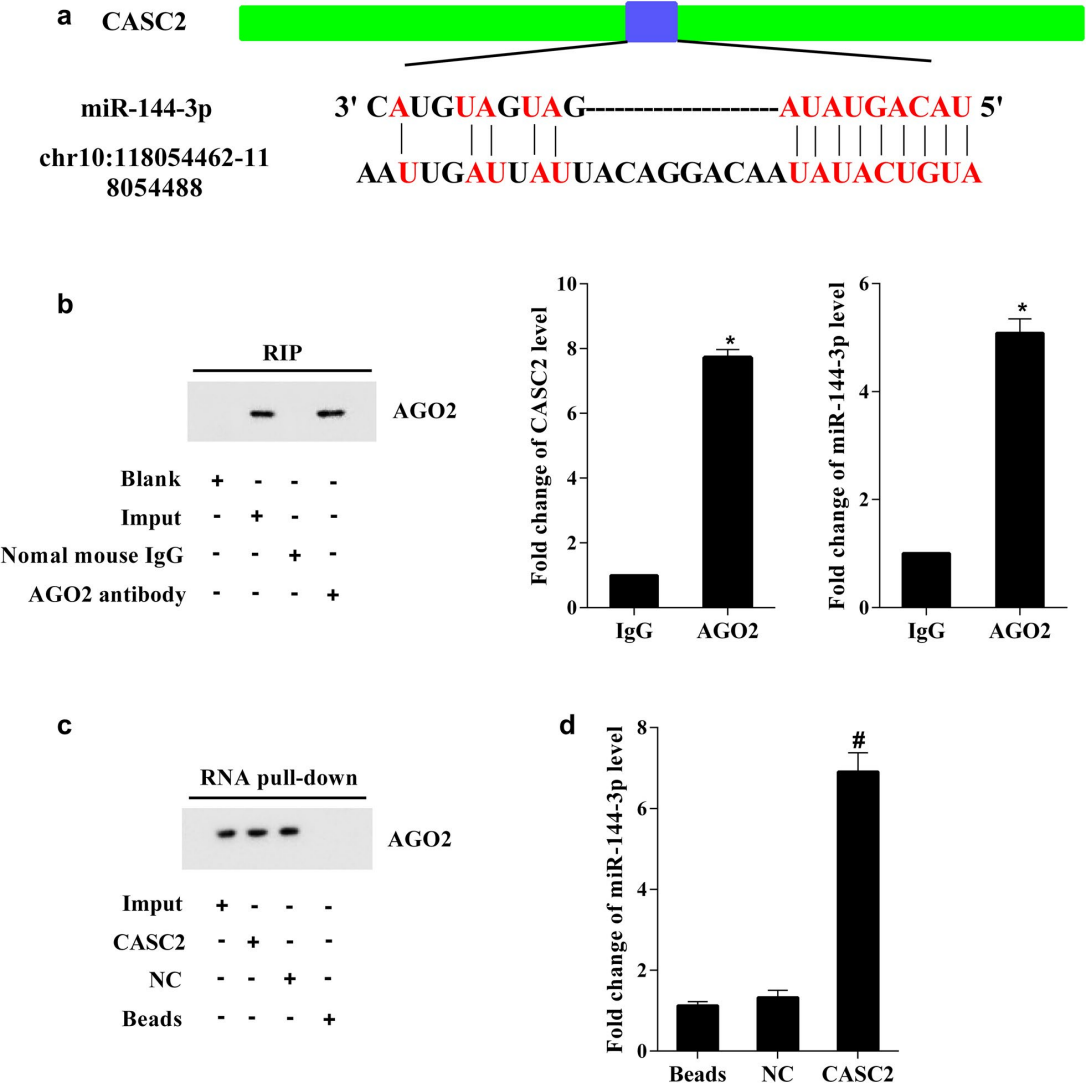
CASC2 functions as a miR-144-3p decoy. **a** Prediction of miR-144-3p binding sites on the CASC2 transcript. **b** CASC2 and miR-144-3p were both existed in AGO2 antibody coprecipitates. **c** The CASC2 binds to the AGO2 protein. **d** The miR-144-3p expression was significantly higher in CASC2 pull-down compound. *P < 0.05 vs IgG, ^#^P < 0.05 vs NC

Based on the results that the expression level of CASC2 was opposite to miR-144-3p, we guessed that CASC2 may function as a miR-144-3p decoy. As Fig. 4a displayed that the predicted positions of miR-144-3p binding sites on the CASC2 transcript, and then RNA immunoprecipitation and RNA pull-down were used to demonstrate their relationship. As shown in Fig. 4b, the CASC2 and miR-144-3p were both existed and obviously increased in AGO2 antibody coprecipitates, which suggested that both of CASC2 and miR-144-3p could bind to AGO2 protein. As shown in Fig. 4c, the AGO2 protein existed in CASC2 drop-down compound, which further confirmed a direct interaction of CASC2 with AGO2. At last, the RT-PCR showed that miR-144-3p expression was significantly increased in CASC2 drop-down compound (Fig. 4d) which further suggested that CASC2 functioned as a miR-144-3p decoy in A549 cells.

### CASC2 controlled AQP1 expression by regulating miR-144-3p

**Fig. 5:**
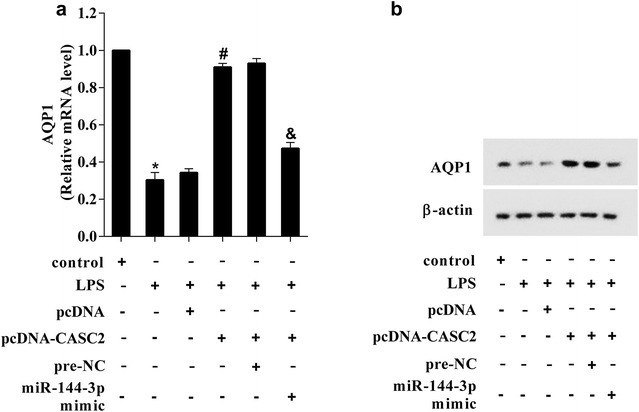
CASC2 controlled AQP1 expression by regulating miR-144-3p. **a** The AQP1 mRNA expression level was decreased in LPS-induced A549 cell. Under LPS induced environment, transfection of pcDNA-CASC2 increased AQP1 mRNA expression level, while co-transfection of pcDNA-CASC2 and miR-144-3p mimic reversed the increased AQP1 mRNA expression level. **b** The AQP1 protein expression level in A549 cell were in accord with the mRNA expression. *P < 0.05 vs pcDNA, ^#^P < 0.05 vs pcDNA-CASC2 + pre-NC, ^&^P < 0.05 vs pcDNA-CASC2 + miR-144-3p mimic

To investigate the effect of CASC2 on AQP1 expression, the A549 cell was induced by LPS and then transfected with pcDNA-CASC2 or co-transfected with pcDNA-CASC2 andmiR-144-3p. As Fig. 5a showed that the AQP1 mRNA expression level was decreased in LPS-induced A549 cell. The A549 cell transfected with pcDNA-CASC2 and then induced by LPS, the results showed that AQP1 mRNA expression level was higher than that in A549 cell transfected with pcDNA. Finally, the A549 cell co-transfected with pcDNA-CASC2 and miR-144-3p mimic and then induced by LPS, and the results showed that AQP1 mRNA expression level was lower than that in A549 cell co-transfected with pcDNA-CASC2 and pre-NC. The western blot results showed in Fig. 5b revealed the same expression changes of AQP1 gene. These data suggested that CASC2 regulated AQP1 mRNA and protein expression via miR-144-3p.

### AQP1 was a target of miR-144-3p

**Fig. 6:**
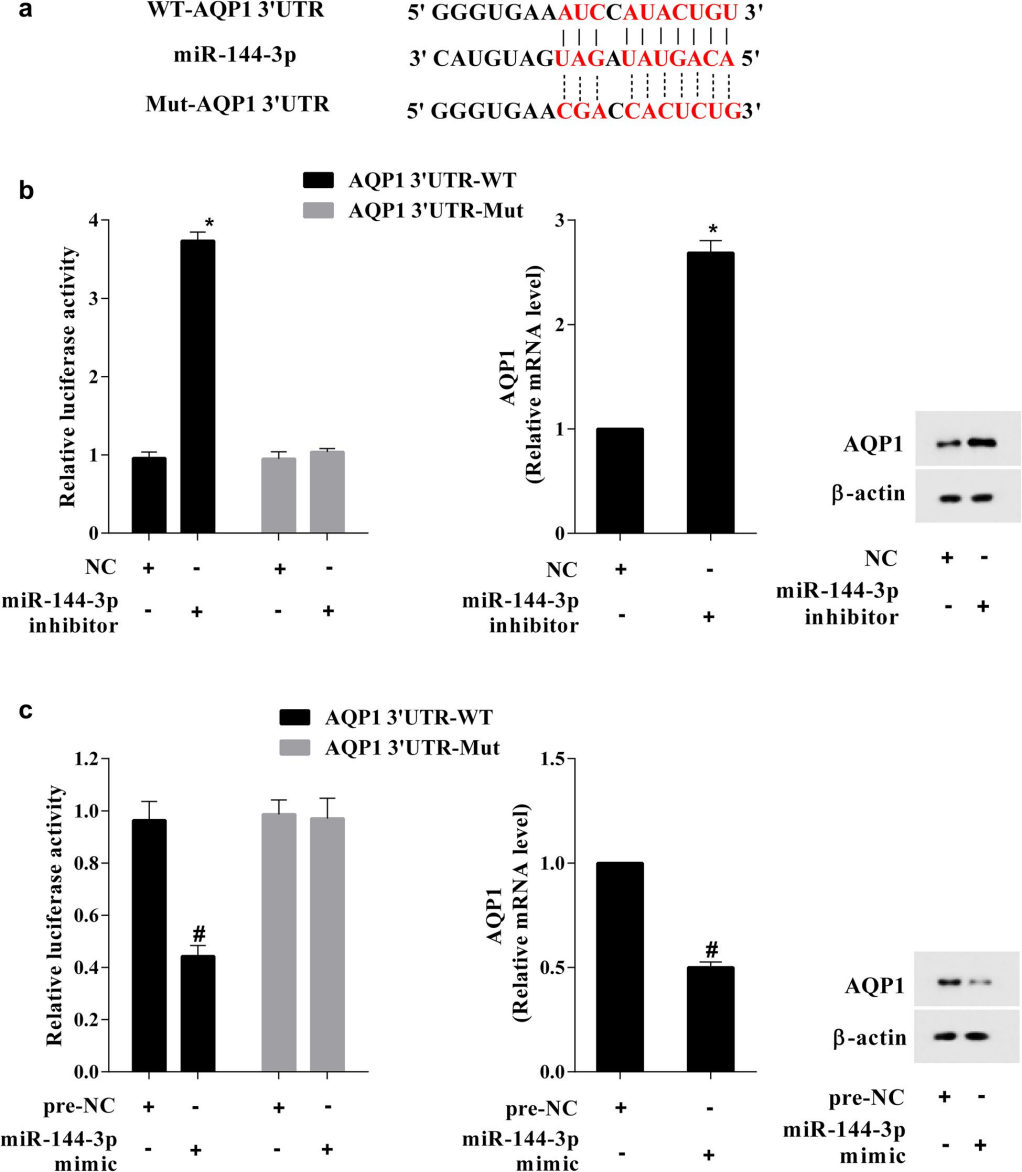
AQP1 was a target of miR-144-3p in A549 cell. **a** According to the predicted binding site of miR-144-3p in AQP1 transcript, we constructed a mutant plasmid of AQP1. **b** The miR-144-3p inhibitor enhanced the luciferase activity in WT-AQP1-transfected A549 cell, while have no impact in MUT-AQP1-transfected A549 cell. Meanwhile, the AQP1 mRNA and protein expression levels were significantly increased in miR-144-3p inhibitor-transfected A549 cells. **c** The miR-144-3p mimic reduced the luciferase activity in WT-AQP1-transfected A549 cell, while have no impact in MUT-AQP1-transfected A549 cell. Meanwhile, the AQP1 mRNA and protein expression levels were significantly decreased in miR-144-3p mimic-transfected A549 cells. *P < 0.05 vs pre-NC, ^#^P < 0.05 vs NC

As AQP1 expression was opposite to miR-144-3p expression in ALI mice and LPS-induced A549 cell, and the AQP1 was predicted to be a target of miR-144-3p (Fig. 6a), so we performed the luciferase reporter assay to verify that whether miR-144-3p could bind to 3′UTR of AQP1. As Fig. 6b showed that the miR-144-3p inhibitor co-transfected with pcDNA-WT-AQP1 or pcDNA-MUT-AQP1, and the result showed that the miR-144-3p inhibitor enhanced the luciferase activity in WT-AQP1-transfected A549 cell, while have no impact in pcDNA-MUT-AQP1-transfected A549 cell. Meanwhile, the AQP1 mRNA and protein expression levels were significantly increased in miR-144-3p inhibitor-transfected A549 cells. In addition, the miR-144-3p mimic reduced the luciferase activity in WT- AQP1-transfected A549 cell, while have no impact in pcDNA-MUT-AQP1- transfected A549 cell. Meanwhile, the AQP1 mRNA and protein expression levels were significantly decreased in miR-144-3p mimic-transfected A549 cells (Fig. 6c). These data showed that miR-144-3p could bind to 3′UTR of AQP1 and regulated AQP1 expression in gene transcription and translation levels.

### LPS induced A549 cell apoptosis by regulating CASC2/miR-144-3p/AQP1 axis

**Fig. 7:**
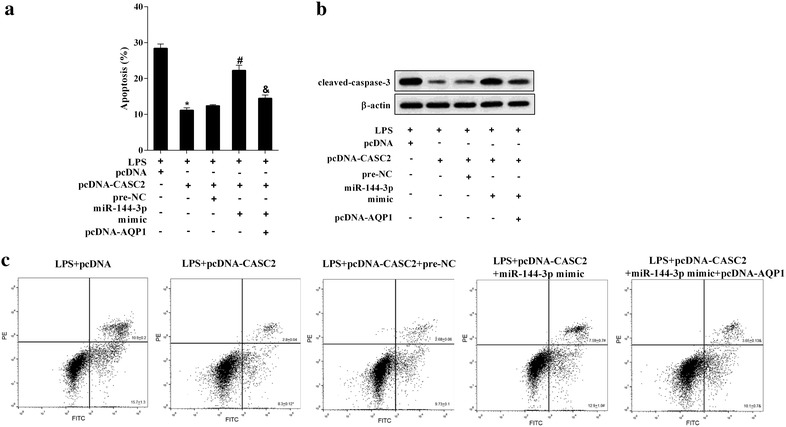
LPS induced A549 cell apoptosis by regulating CASC2/miR-144-3p/AQP1 axis. The A549 cell was transfected or co-transfected of pcDNA-CASC2, miR-144-3p mimic and pcDNA-AQP1 and induced by LPS. And then the cell apoptosis was detected by MTT, and demonstrated by caspase-3 expression. **a** The inhibited cell apoptosis by pcDNA-CASC2 was reversed by miR-144-3p mimic, while which was reversed again by pcDNA-AQP1. **b** The caspase-3 expression by western blot. **c** A549 cell apoptosis in different groups using flow cytometry assay. *P < 0.05 vs pcDNA, ^#^P < 0.05 vs pre-NC, ^&^P < 0.05 vs miR-144-3p mimic

To investigate whether CASC2/miR-144-3p/AQP1 axis was involved the LPS-induced A549 cell apoptosis, the A549 cell induced by LPS, and transfection or co-transfection of pcDNA-CASC2, miR-144-3p mimic and pcDNA-AQP1. As showed in Fig. 7, pcDNA-CASC2 transfection reversed LPS-induced cell apoptosis, while miR-144-3p mimic reversed pcDNA-CASC2-reduced cell apoptosis. In addition, pcDNA-AQP1 reversed again the effect of pcDNA-CASC2 combined miR-144-3p mimic on cell apoptosis. The caspase-3 expression in Fig. 7b further revealed that LPS induced A549 cell apoptosis through regulating CASC2/miR-144-3p/AQP1 axis.

### Overexpression of CASC2 alleviated LPS-induced acute lung injury in mice

**Fig. 8:**
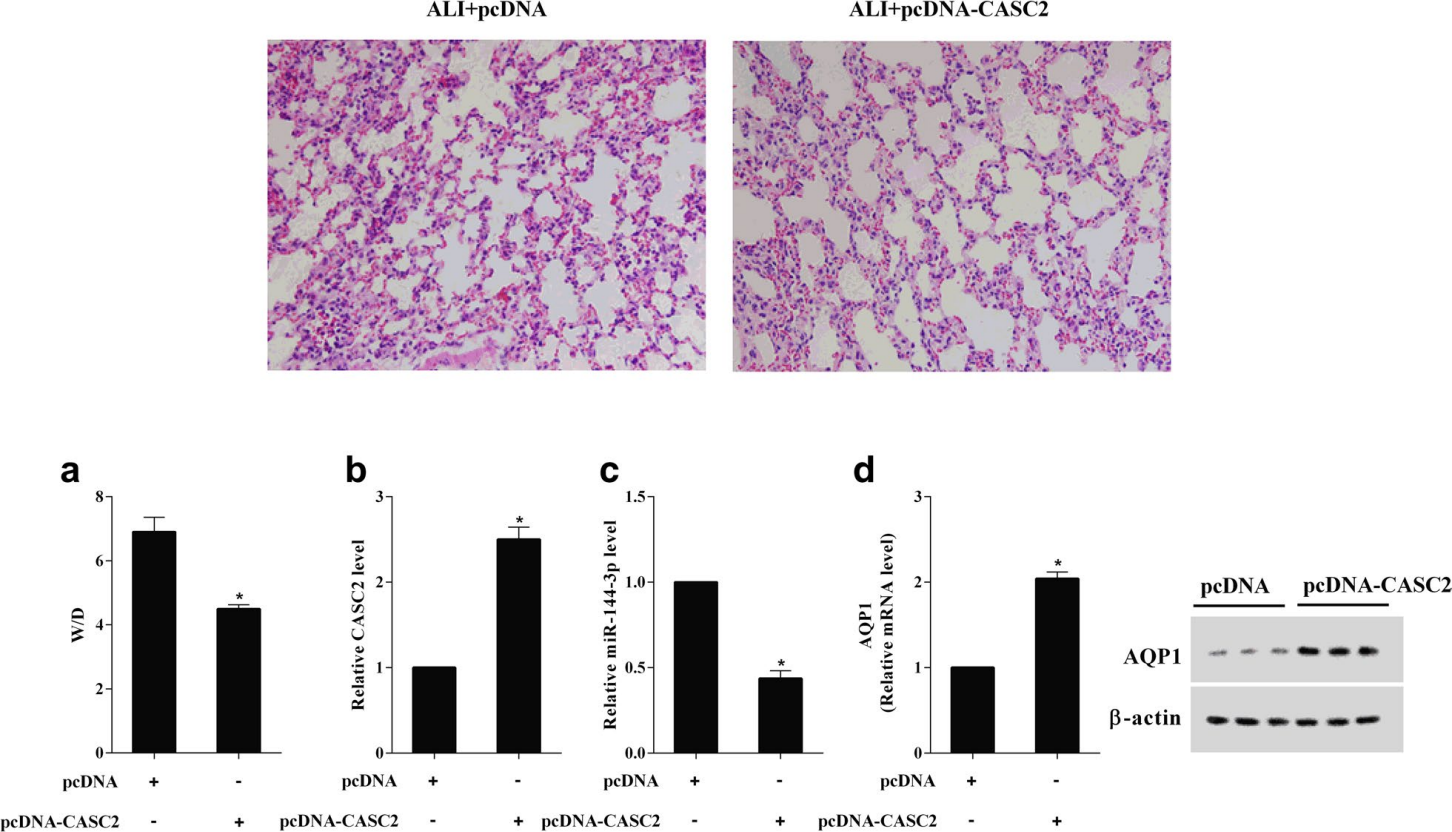
Overexpression of CASC2 alleviated LPS-induced acute lung injury in mice. **a** The lung wet–dry weight ratio (W/D) was significantly reduced in Lv-CASC2 group. **b** The increased CASC2 expression in lung tissues of Lv-CASC2-injected mice. **c** The decreased miR-144-3p expression in lung tissues of Lv-CASC2-injected mice. **d** The increased CASC2 mRNA and protein expression in lung tissues of Lv-CASC2-injected mice

To study the effect of overexpression CASC2 on alleviating acute lung injury in mice, the mice were injected with lentivirus-expressing CASC2 (pcDNA-CASC2) or the control lentivirus (pcDNA) by tail vein, and then treated with LPS. H&E staining of the lung tissue of ALI mice injected with pcDNA and ALI mice injected with pcDNA-CASC2 was shown in Fig. 1. As Fig. 8a showed that the lung wet–dry weight ratio (W/D) in pcDNA-CASC2-injected mice was significantly lower than in pcDNA-injected mice, which indicated that overexpression CASC2 could alleviate acute lung injury in mice. In addition, the CASC2, miR-144-3p and AQP1 expression in lung tissues were also analyzed by RT-qPCR and western blot. The CASC2 expression was obviously increased, while the miR-144-3p expression was obviously decreased in lung tissues of pcDNA-CASC2-injected mice (Fig. 8b, c). The AQP1 mRNA and protein expression were both significantly increased in lung tissues of pcDNA-CASC2-injected mice (Fig. 8d). These results implied that overexpression of CASC2 alleviated LPS-induced acute lung injury in mice.

## Discussion

ALI is a serious disease with high morbidity and mortality rate. Although there are effective strategies for treatment of ALI, a widely accepted specific pharmacotherapy has not yet established [16]. Conventional research on ALI often involved investigations on gene expression and regulation, specific protein or protein related signaling pathways, etc. The discovery of lncRNA reveals a new insight on the pathogenesis of ALI which will open a new era for lung researchers to develop specific effective therapeutics and diagnostics. A recent study have identified a highly up-regulated mammalian lncRNA, FOXD3-AS1, in the setting of hyperoxia/ROS-induced lung injury [17]. Another study demonstrated that the lncRNA-NANCI was decreased in hyperoxia-induced lung injury mice and involved in the development of immature lung tissues [18]. Lipopolysaccharide (LPS) is one of the major factors that induce acute lung injury and lung endothelial apoptosis. To date, the study of lncRNA on LPS-induced ALI has not been reported. The novel lncRNA CASC2 is located on chromosome 10 in humans and has been characterized as a tumor suppressor in human malignancies [19]. In this study, we found that LPS induced a downregulated CASC2 in mice lung tissue and lung epithelial cell (Figs. 1a and 2a). Overexpression of CASC2 inhibited LPS-induced lung epithelial cell apoptosis (Fig. 3), and reduced lung wet–dry weight ratio in LPS-induced ALI mice (Fig. 8). Thus, CASC2 played an important role in LPS-induced ALI.

Aquaporin-1 water channels are membrane proteins that control the permeability of endothelial and epithelial barriers by facilitating water movement across cell membranes. In addition to be responsible for the high vascular permeability and interstitial fluid pressure in tumors of the brain, colon, breast and pancreas, AQP1 was reported to play a key role in the pathogenesis of ALI. This study revealed that AQP1 expression was decreased in LPS-induced ALI mice lung tissue and lung epithelial cell (Figs. 1c and 2c), and overexpression of AQP1 reduced cell apoptosis, which was identical with those reported in literature [7, 8]. There are a few miRNA, such as miR-320 [20], miR-666 and miR-708 [21], which have identified as potential modulator of AQP1. In this study, the miR-144-3p expression was opposite to AQP1 expression in LPS-induced ALI mice lung tissue and lung epithelial cell (Figs. 1b and 2b), and the luciferase reporter assay revealed that AQP1 was a target of miR-144-3p (Fig. 6). Previous study showed that the miR-144-3p expression was increased in A549 cell, and miR-144-3p mimic suppressed cell proliferation [22]. In the present study, we found that miR-144-3p expression was increased in LPS-induced ALI mice lung tissue and A549 cell, and miR-144-3p mimic promoted cell apoptosis. The results presented in current study are in agreement with the previous study. Further, we found that pcDNA-AQP1 reversed the effect of miR-144-3p mimic on cell apoptosis. Thus, miR-144-3p participated in LPS-induced ALI and lung epithelial cells apoptosis by regulating AQP1.

Studies have also shown that lncRNAs functioned as competing endogenous RNAs or as molecular sponges in modulating the concentration and biological functions of miRNAs [23]. For example, Fan et al. [24] reported the ability of PTCSC3 to bind hsa-miR-574-5p as ceRNA. H19 triggers EMT progression by binding miR-138-5p and miR-200a-3p, antagonizing their functions and leading to the increase of their endogenous targets [25]. LncRNA CASC2 have been demonstrated as playing crucial regulatory roles in a few of cancers, and functioned as endogenous RNA by sponging miRNAs, such as miR-18a [26], miR-367 [27], miR-21 [28], etc., so whether the CASC2 could function as a miR-144-3p decoy was verified by the RNA immunoprecipitation (RIP) and RNA pull-down assay in this study. And then, the mechanism of CASC2 on AQP1 expression was investigated. Co-transfection with pcDNA-CASC2 and miR-144-3p mimic reversed the pcDNA-CASC2-upregulated AQP1 expression (Fig. 5), which fully demonstrated CASC2 controlled AQP1 expression by regulating miR-144-3p. At last, whether CASC2/miR-144-3p/AQP1 axis was involved the LPS-induced lung epithelial cell apoptosis was investigated. The pcDNA-CASC2 transfection reversed LPS-induced cell apoptosis, miR-144-3p mimic again reversed pcDNA-CASC2-reduced cell apoptosis, and pcDNA-AQP1 reversed again the effect of pcDNA-CASC2 combined miR-144-3p mimic on cell apoptosis, which implied LPS induced A549 cell apoptosis through regulating CASC2/miR-144-3p/AQP1 axis.

## Conclusions

In summary, the expression of CASC2 was decreased, while miR-144-3p was increased in LPS-induced ALI mice and lung epithelial cell. Further, CASC2 and AQP1 were targets of miR-144-3p and CASC2 can indirectly regulate AQP1 expression via miR-144-3p. Moreover, CASC2 regulated A549 cell apoptosis through regulating miR-144-3p and its target, AQP1. The results concerning CASC2 functionality and molecular mechanisms relevant to LPS-induced ALI provided a new perspective for lncRNA-directed therapeutic target for LPS-induced ALI.
